# Poly[(μ_4_-tetra­zole-1-acetato-κ^4^ *N* ^3^:*N* ^4^:*O*:*O*′)silver(I)]

**DOI:** 10.1107/S1600536810001236

**Published:** 2010-01-16

**Authors:** Shi-Jie Li, Hao Wang, Wen-Dong Song, Shi-Wei Hu, Pei-Wen Qin

**Affiliations:** a College of Food Science and Technology, Guang Dong Ocean University, Zhanjiang 524088, People’s Republic of China; b College of Science, Guang Dong Ocean University, Zhanjiang 524088, People’s Republic of China; c College of Agriculture, Guang Dong Ocean University, Zhanjiang 524088, People’s Republic of China

## Abstract

In the title complex, [Ag(C_3_H_3_N_4_O_2_)]_*n*_, the Ag^I^ atom is four-coordinated in a slightly distorted tetra­hedral coordination geometry by two N atoms from two tetra­zole-1-acetate (tza) ligands and two O atoms from the other two tza ligands. The tza ligand bridges two Ag atoms through the carboxyl­ate O atoms and simultaneously binds to the other two Ag atoms through the tetra­zole N atoms, forming a two-dimensional network parallel to (100).

## Related literature

For the diverse coordination modes and potential applications of metal complexes with tetra­zole derivatives, see: Stagni *et al.* (2006 ▶); Ye *et al.* (2006 ▶).
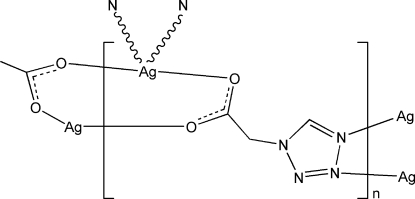


## Experimental

### 

#### Crystal data

[Ag(C_3_H_3_N_4_O_2_)]
*M* *_r_* = 234.96
Triclinic, 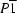

*a* = 5.1584 (10) Å
*b* = 7.7805 (16) Å
*c* = 7.8711 (16) Å
α = 109.40 (3)°
β = 98.87 (3)°
γ = 104.85 (3)°
*V* = 277.92 (14) Å^3^
*Z* = 2
Mo *K*α radiation
μ = 3.56 mm^−1^
*T* = 293 K
0.25 × 0.23 × 0.21 mm

#### Data collection

Rigaku/MSC Mercury CCD diffractometer
Absorption correction: multi-scan (*REQAB*; Jacobson, 1998 ▶) *T*_min_ = 0.470, *T*_max_ = 0.522
2722 measured reflections
1267 independent reflections
1150 reflections with *I* > 2σ(*I*)
*R*_int_ = 0.056

#### Refinement

*R*[*F* ^2^ > 2σ(*F* ^2^)] = 0.062
*wR*(*F* ^2^) = 0.177
*S* = 1.23
1267 reflections
92 parameters
H-atom parameters constrained
Δρ_max_ = 2.15 e Å^−3^
Δρ_min_ = −0.97 e Å^−3^

### 

Data collection: *CrystalStructure* (Rigaku/MSC, 2002 ▶); cell refinement: *CrystalStructure*; data reduction: *CrystalStructure*; program(s) used to solve structure: *SHELXS97* (Sheldrick, 2008 ▶); program(s) used to refine structure: *SHELXL97* (Sheldrick, 2008 ▶); molecular graphics: *ORTEPII* (Johnson, 1976 ▶) and *DIAMOND* (Brandenburg, 1999 ▶); software used to prepare material for publication: *SHELXL97*.

## Supplementary Material

Crystal structure: contains datablocks I, global. DOI: 10.1107/S1600536810001236/hy2270sup1.cif

Structure factors: contains datablocks I. DOI: 10.1107/S1600536810001236/hy2270Isup2.hkl

Additional supplementary materials:  crystallographic information; 3D view; checkCIF report

## Figures and Tables

**Table 1:** Selected bond lengths (Å)

Ag1—O1	2.330 (7)
Ag1—O2^i^	2.282 (7)
Ag1—N3^ii^	2.494 (9)
Ag1—N4^iii^	2.442 (8)

Symmetry codes: (i) 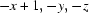; (ii) 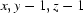; (iii) 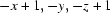.

**Fig. 1.**
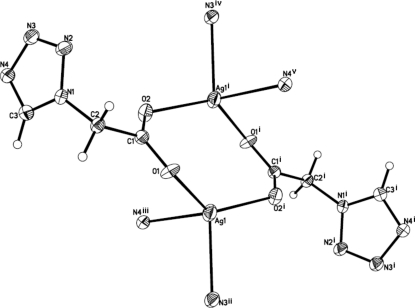
The asymmetric unit of the title compound, with symmetrically related atoms to complete the Ag coordination. Displacement ellipsoids are drawn at the 30% probability level. [Symmetry codes: (i) 1-x, -y, -z; (ii) x, -1+y, -1+z; (iii) 1-x, -y, 1-z; (iv) 1-x, 1-y, 1-z; (v) x, y, -1+z.]

**Fig. 2.**
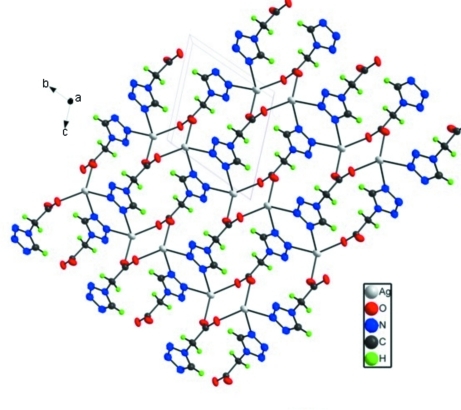
A view of the layer structure of the title compound.

## supplementary crystallographic information

### Comment

In recent years, organic ligands with a tetrazole functional group have been greatly used in coordination chemistry for construction of metal-organic frameworks due to their diverse coordination modes and potential applications in varied fields (Stagni *et al.*, 2006; Ye *et al.*, 2006). The reaction of tetrazole-1-acetic acid (Htza) with AgNO_3_ in an alkaline aqueous solution yielded a new Ag^I^ coordination polymer, whose crystal structure is reported here.

In the title complex, the Ag^I^ atom is four-coordinated in a slightly distorted tetrahedral coordination geometry by two N atoms and two O atoms from four different tza ligands (Table 1), as illustrated in Fig. 1. The adjacent Ag^I^ atoms are co-bridged by tza liands. The tza ligand acts as a tetradentate ligand, bridging two Ag atoms through its carboxylate O atoms, while simultaneously binding to the other two Ag atoms through two N atoms of the tetrazole group, forming a two-dimensional network parallel to (1 0 0).

### Experimental

A mixture of AgNO_3_ (0.073 g, 0.5 mmol) and Htza (0.990 g, 0.5 mmol) in 15 ml of H_2_O solution was sealed in an autoclave equipped with a Teflon liner (20 ml) and then heated at 373 K for 4 d. Crystals of the title compound were obtained by slow evaporation of the solvent at room temperature.

### Refinement

H atoms were placed at calculated positions and treated as riding on the parent C atoms, with C—H = 0.93 (CH) and 0.97 (CH_2_) Å and with *U*_iso_(H) = 1.2*U*_eq_(C). The highest residual electron density was found 1.40 Å from N4 and the deepest hole 1.12 Å from Ag1.

### Crystal data

**Table:**

[Ag(C_3_H_3_N_4_O_2_)]	*Z* = 2
*M**_r_* = 234.96	*F*(000) = 224
Triclinic, *P*1	*D*_x_ = 2.808 Mg m^−^^3^
Hall symbol: -P 1	Mo *K*α radiation, λ = 0.71073 Å
*a* = 5.1584 (10) Å	Cell parameters from 3600 reflections
*b* = 7.7805 (16) Å	θ = 1.4–28°
*c* = 7.8711 (16) Å	µ = 3.56 mm^−^^1^
α = 109.40 (3)°	*T* = 293 K
β = 98.87 (3)°	Block, blue
γ = 104.85 (3)°	0.25 × 0.23 × 0.21 mm
*V* = 277.92 (14) Å^3^	

### Data collection

**Table:**

Rigaku/MSC Mercury CCD diffractometer	1267 independent reflections
Radiation source: fine-focus sealed tube	1150 reflections with *I* > 2σ(*I*)
graphite	*R*_int_ = 0.056
ω scans	θ_max_ = 27.5°, θ_min_ = 3.2°
Absorption correction: multi-scan (REQAB; Jacobson, 1998)	*h* = −6→6
*T*_min_ = 0.470, *T*_max_ = 0.522	*k* = −9→10
2722 measured reflections	*l* = −10→9

### Refinement

**Table:**

Refinement on *F*^2^	Secondary atom site location: difference Fourier map
Least-squares matrix: full	Hydrogen site location: inferred from neighbouring sites
*R*[*F*^2^ > 2σ(*F*^2^)] = 0.062	H-atom parameters constrained
*wR*(*F*^2^) = 0.177	*w* = 1/[σ^2^(*F*_o_^2^) + (0.0519*P*)^2^ + 3.2858*P*] where *P* = (*F*_o_^2^ + 2*F*_c_^2^)/3
*S* = 1.23	(Δ/σ)_max_ < 0.001
1267 reflections	Δρ_max_ = 2.15 e Å^−^^3^
92 parameters	Δρ_min_ = −0.97 e Å^−^^3^
0 restraints	Extinction correction: *SHELXL97* (Sheldrick, 2008), Fc^*^=kFc[1+0.001xFc^2^λ^3^/sin(2θ)]^-1/4^
Primary atom site location: structure-invariant direct methods	Extinction coefficient: 0.052 (15)

### Fractional atomic coordinates and isotropic or equivalent isotropic displacement parameters (Å^2^)

**Table:**

	*x*	*y*	*z*	*U*_iso_*/*U*_eq_	
Ag1	0.61189 (17)	−0.19436 (12)	−0.05077 (10)	0.0387 (5)	
O1	0.9281 (15)	0.0639 (10)	0.2082 (10)	0.0365 (16)	
O2	0.6195 (14)	0.1949 (12)	0.3227 (10)	0.0360 (16)	
N1	0.9120 (16)	0.3243 (11)	0.6770 (10)	0.0277 (16)	
N2	0.872 (2)	0.4946 (12)	0.7248 (12)	0.0376 (19)	
N3	0.7161 (19)	0.4973 (12)	0.8396 (12)	0.0363 (19)	
N4	0.656 (2)	0.3322 (13)	0.8697 (12)	0.0351 (18)	
C1	0.8444 (18)	0.1645 (13)	0.3374 (12)	0.0265 (17)	
C2	1.0509 (18)	0.2615 (13)	0.5323 (12)	0.0267 (17)	
H2A	1.1368	0.1710	0.5545	0.032*	
H2B	1.1964	0.3719	0.5373	0.032*	
C3	0.781 (2)	0.2254 (15)	0.7646 (14)	0.034 (2)	
H3	0.7775	0.1022	0.7545	0.041*	

### Atomic displacement parameters (Å^2^)

**Table:**

	*U*^11^	*U*^22^	*U*^33^	*U*^12^	*U*^13^	*U*^23^
Ag1	0.0430 (6)	0.0441 (6)	0.0259 (5)	0.0145 (4)	0.0071 (3)	0.0105 (3)
O1	0.033 (3)	0.031 (3)	0.034 (4)	0.013 (3)	0.010 (3)	−0.003 (3)
O2	0.028 (3)	0.055 (4)	0.029 (3)	0.020 (3)	0.008 (3)	0.016 (3)
N1	0.033 (4)	0.030 (4)	0.021 (3)	0.011 (3)	0.008 (3)	0.009 (3)
N2	0.051 (5)	0.027 (4)	0.033 (4)	0.013 (4)	0.017 (4)	0.008 (3)
N3	0.045 (5)	0.030 (4)	0.030 (4)	0.010 (4)	0.012 (4)	0.008 (3)
N4	0.048 (5)	0.034 (4)	0.030 (4)	0.020 (4)	0.017 (4)	0.014 (3)
C1	0.026 (4)	0.028 (4)	0.025 (4)	0.010 (3)	0.005 (3)	0.009 (3)
C2	0.023 (4)	0.032 (4)	0.022 (4)	0.010 (3)	0.003 (3)	0.007 (3)
C3	0.041 (5)	0.033 (5)	0.032 (5)	0.014 (4)	0.013 (4)	0.015 (4)

### Geometric parameters (Å, °)

**Table:**

Ag1—O1	2.330 (7)	N1—C2	1.453 (11)
Ag1—O2^i^	2.282 (7)	N2—N3	1.297 (12)
Ag1—N3^ii^	2.494 (9)	N3—N4	1.350 (12)
Ag1—N4^iii^	2.442 (8)	N4—C3	1.331 (13)
O1—C1	1.270 (11)	C1—C2	1.540 (12)
O2—C1	1.238 (11)	C2—H2A	0.9700
N1—C3	1.324 (12)	C2—H2B	0.9700
N1—N2	1.331 (12)	C3—H3	0.9300
			
O2^i^—Ag1—O1	129.2 (3)	C3—N4—N3	105.1 (8)
O2^i^—Ag1—N4^iii^	118.7 (3)	C3—N4—Ag1^iii^	117.8 (6)
O1—Ag1—N4^iii^	95.0 (3)	N3—N4—Ag1^iii^	137.0 (6)
O2^i^—Ag1—N3^ii^	102.2 (3)	O2—C1—O1	127.3 (9)
O1—Ag1—N3^ii^	118.0 (3)	O2—C1—C2	117.2 (8)
N4^iii^—Ag1—N3^ii^	86.0 (3)	O1—C1—C2	115.5 (8)
C1—O1—Ag1	120.6 (6)	N1—C2—C1	111.1 (7)
C1—O2—Ag1^i^	121.2 (6)	N1—C2—H2A	109
C3—N1—N2	109.2 (8)	C1—C2—H2A	109
C3—N1—C2	128.9 (8)	N1—C2—H2B	109
N2—N1—C2	121.4 (8)	C1—C2—H2B	109
N3—N2—N1	106.3 (8)	H2A—C2—H2B	108
N2—N3—N4	111.0 (8)	N1—C3—N4	108.4 (9)
N2—N3—Ag1^iv^	112.1 (6)	N1—C3—H3	126
N4—N3—Ag1^iv^	136.9 (6)	N4—C3—H3	126

Symmetry codes: (i) −*x*+1, −*y*, −*z*; (ii) *x*, *y*−1, *z*−1; (iii) −*x*+1, −*y*, −*z*+1; (iv) *x*, *y*+1, *z*+1.
